# The Epidemiology of African Swine Fever, Its Complexity and the Requirement for Multiple Solution Approaches

**DOI:** 10.3390/ani10101900

**Published:** 2020-10-16

**Authors:** Katja Schulz, Anette Boklund

**Affiliations:** 1 Institute of Epidemiology, Friedrich-Loeffler-Institut, Federal Research Institute for Animal Health, Südufer 10, 17493 Greifswald, Insel Riems, Germany; 2 Department of Veterinary and Animal Sciences, University of Copenhagen, 1870 Frederiksberg C, Denmark; anebo@sund.ku.dk

## 1. Knowledge

Searching for the term “African swine fever” (ASF) in the title of publications in the Web of Science, PubMed and Scopus during the period of 1955–2020 resulted in an average of 2009 detected articles. This shows that the scientific interest in ASF is consistently high. This is certainly due to the ongoing global spread of ASF and the knowledge gaps that still exist [1]. After the successful elimination of ASF from the Iberian Peninsula in 1995, ASF almost disappeared from the European continent [2]. However, the disease has been endemic in Sardinia since 1978, despite several attempted elimination programs. It has been hypothesized that mainly social structures and traditions, like the keeping of free-ranging pigs, and slaughters for self-consumption, pose a significant risk for the presence of ASF on the island and decrease the effectiveness of standard control measures [3]. However, there is hope that the disease is close to eradication now. Since 2007, when ASF emerged in Georgia, the disease has been spreading constantly, affecting eleven countries of the European Union to date. Although domestic pig holdings are also regularly affected, most infections occur in wild boar [4]. However, several studies have shown that an infected wild boar population in close vicinity to domestic pig farms poses a risk of transmitting the disease into domestic pig holdings, in particular backyard holdings [5,6,7]. Due to these findings and to the potential enormous economic consequences that an ASF outbreak entails, it is vital to increase the current knowledge on ASF to fight the disease more efficiently.

## 2. Theses

In contrast to conventional assumptions that ASF is a highly contagious disease, which rushes through a wild boar or pig population without leaving any survivors, a recent review demonstrated the rather difficult definition of the speed of transmission of ASF and corresponding parameters [8]. In addition to these uncertainties, Blome, et al. [1] identified the current most pressing knowledge gaps in the different fields concerning ASF. So far, no scientific proof has been found for the theory that surviving wild boar play a role as virus carriers in the transmission of ASF [9]. By contrast, seropositive animals survived the infection and were able to eliminate the virus [10]. Accordingly, it has been hypothesized that an increase in seropositive wild boar among all sampled wild boar might indicate a subsiding epidemic [11,12,13]. However, studies that prove the lack of infectiousness of these animals or the possibility of reactivation of ASFV are still missing. 

On several occasions, it has been thought that ASF was introduced by human activities. Swill feeding, the unsafe disposal of pork leftovers or insufficient hygiene measures during hunting represent only a few examples for human activities that can lead to virus introduction. To study the influence of human behavior and wild boar biology on the epidemiology of ASF, it is of utmost importance to include a transdisciplinary approach in the joint fight against this animal disease. 

## 3. Facts

Many biological parameters regarding ASFV are well studied and generally known [14]. However, the apparently, unstoppable spread of ASF and the associated threat for the global economy requires inter- and transdisciplinary research to combat the disease effectively. Thus, not only epidemiologists, virologists, immunologists and pathologists, but also social scientists, mathematicians and wild-life biologists should combine their knowledge and expertise in order to achieve the goal of controlling ASF.
